# Time trends in non-suicidal self-injury, disordered eating, and mental health problems between 2007 and 2025: a comparison of two Swedish adolescent cohorts

**DOI:** 10.1007/s00787-026-03105-4

**Published:** 2026-06-22

**Authors:** Benjamin Claréus, Lars-Gunnar Lundh, Margit Wångby-Lundh, Jonas Bjärehed, Daiva Daukantaitė

**Affiliations:** 1 Department of Psychology, Kristianstad University, Kristianstad, 291 88 Sweden; 2 Department of Psychology, Lund University, Lund, 221 00 Sweden

**Keywords:** Time trends, Prevalence, Non-suicidal self-injury, Disordered eating, Mental health, Adolescence

## Abstract

Reports suggest rising trends in non-suicidal self-injury (NSSI) and disordered eating (DE) amid worsening adolescent mental health; however, the use of single-item measures obscures these findings. Moreover, there are no studies of time trends in NSSI/DE co-occurring with other mental health problems. The present study used multi-item measures to examine time trends in the prevalence of NSSI, DE, other mental health problems, and their co-occurrence in two separate school cohorts (2007/2008; 2024/2025) in the same Swedish municipality. Clinically relevant cutoffs were applied to self-report measures of NSSI (revised Deliberate Self-Harm Inventory), DE (Risk Behaviour related to Eating Disorders; girls only), and mental health problems (MHP; Strengths and Difficulties Questionnaire – self-report). Analyses included gender-stratified mixed models (time trends per outcome) and binominal generalized log-linear models (time trends in NSSI/DE×MHP concurrence). Response rates were ≈ 90% in 2007/2008 (*N* = 987–992; *M*_age_ = 13.71–14.76) and ≈ 80% in 2024/2025 (*N* = 810–836; *M*_age_ = 13.71–14.79). Among girls, there were threefold increases in the prevalence of repetitive NSSI and high-risk DE combined with other mental health problems (NSSI + MHP from 6.6 to 21.0%; DE + MHP from 2.7 to 8.7%). The prevalence of repetitive NSSI or high-risk DE without co-occurring mental health problems did not change. Among boys, there was no increase in repetitive NSSI with/without other mental health problems. The rising trends in the prevalence of NSSI and DE among girls indicate worsening mental health, suggesting a need for increased attention and action from the healthcare system.

**Supplementary Information:**

The online version contains supplementary material available at https://doi.org/10.1007/s00787-026-03105-4.

Concerns about adolescents’ mental health have intensified in recent decades in the Global North. Large international monitoring programs such as the Global Burden of Disease Study [1], Health Behaviour in School-aged Children Survey [2], and Gallup World Poll [3] report rising rates of internalizing problems (anxiety, depression, general psychological complaints) in adolescents, particularly among girls. However, such programs do not investigate self-reported self-destructive behaviour such as non-suicidal self-injury (NSSI; intentional self-injury without suicidal intent, e.g., cutting) and disordered eating (DE; e.g., restrictive dieting, binge eating, or purging), which can both signal and intensify underlying distress; as such, less is known about the time trends of these harmful behaviours. NSSI and DE typically emerge during adolescence and, if they become recurrent and persistent, are linked to increased risk of mental health problems enduring into adulthood [4, 5]. 

Methodologically sound, up-to-date studies of time trends in self-reported NSSI and DE are scarce. Although studies from Finland (2014–2023) [6], Sweden (2011–2021) [7], and the United Kingdom (2005–2015) [8] suggest that the lifetime prevalence of NSSI increased over 10-year periods, the measurement validity of these studies is compromised by their use of single questions with two [7, 8] or three [6] response categories. As to DE, the evidence is inconsistent. With single-item measures, increases during the past decades have been reported from Australia [9], China [10], the United Kingdom [8, 11], and United States [12]. However, time-trend research with validated measures from Finland (1998–2018) [13] and Germany (2003–2017) [14] suggests decreases or statistically non-significant changes.

In view of the methodological limitations and inconsistent findings on time trends, we call for updated prevalence estimates of NSSI and DE based on validated, conceptually coherent measures that encode the dimensionality, frequency, and severity of these behaviours. Without such metrics, important nuances in symptom burden are obscured, undermining both clinical decision-making and research validity [15]. 

There is also little research on time trends in the prevalence of NSSI or DE co-occurring with mental health problems. Such research is important for several reasons. First, it is important that we understand to what extent trends in NSSI and DE represent increases in clinically significant problems or another kind of phenomenon. For instance, rising rates of NSSI or DE without other mental health problems might reflect changing social norms or subcultures that normalize these behaviours among adolescents.

Second, there is ongoing discussion about the status of NSSI in the spectrum of psychopathology. During the development of the fifth edition of the Diagnostic and Statistical Manual of Mental Disorders (DSM-5) [16], it was suggested that NSSI be seen as a separate disorder with its own diagnostic criteria. However, because of insufficient empirical evidence, NSSI disorder (NSSI-D) was included merely as a “condition requiring further research” in Section III of the DSM-5. Recent research has shown that NSSI-D as a stand-alone diagnosis (i.e., without comorbidity) is rare and characterized by limited emotional distress and low stability [17]. Lengel et al. [18] offered an alternative to conceptualizing NSSI-D as a separate disorder with its own criteria and high comorbidity: namely, NSSI as a clinical specifier for other mental disorders. In the present study, we focus not on NSSI-D but on repetitive NSSI (which is one of several criteria for the suggested diagnosis of NSSI-D), while raising the important question about time trends in the prevalence of NSSI, both stand-alone and in combination with other mental health problems. In other words: if the prevalence of NSSI has increased over the past few decades, is it in combination with other co-occurring mental health problems or independently of these?

Third, analysing time trends in the prevalence of NSSI and DE with co-occurring mental health problems may be relevant to the prevalence inflation hypothesis. According to this hypothesis, increased mental health literacy may lead not only to improved recognition of mental health problems but also to a tendency to pathologize common/normal psychological experiences [19]. It may be argued that self-reported experiences of distress are more subject to overpathologization than are self-reported behaviours, as behaviours have a more “objective” quality than experiences of distress. Therefore, if the prevalence of mental health problems does in fact increase in combination with behavioural problems such as NSSI or DE, this is less likely attributable to an increased tendency to overpathologize normal experiences.

In the present study, we addressed time trends in the prevalence of (1) NSSI, DE, and mental health problems separately and (2) NSSI or DE in combination with other mental health problems. In addition, we explored (3) trends in the strength of interconnectivity of NSSI and DE with other mental health problems. We analysed data from two Swedish population-representative adolescent cohorts separated by 17 years. The focus was on the proportions of adolescents who passed clinically relevant cutoffs for (i) repetitive NSSI, (ii) high-risk DE, and (iii) mental health problems.

## Methods

### Setting

Data were collected within the same medium-sized Swedish municipality during two periods, 2007/2008 (Period 1) and 2024/2025 (Period 2). In each period, the study included all regular school students in the municipality in Grades 7 and 8 (baseline: 2007, 2024) and again one year later in Grades 8 and 9 (follow-up: 2008, 2025). Data were collected as part of the Lund University project “Självkänsla och Livsituation” (SoL).

In Sweden, a municipality is the basic unit of local government. Municipalities are responsible for providing services such as education, utilities, and social services across a defined territory encompassing both urban and rural areas. Based on the definition of foreign background from Statistics Sweden (i.e., being born in Sweden by parents born outside Sweden or being born outside Sweden with at least one parent born abroad) [20], the number of inhabitants with a foreign background in the targeted municipality increased, from 17.2% to 25.1% (cf. 17.3% to 27.5% in Sweden overall). The yearly mean income among municipal inhabitants aged 20–64 years was 4.7% lower than in Sweden overall in 2007, and this gap increased to 5.7% in 2023.

### Measures

Gender (*girl*, *boy*; in 2024/2025, *other/prefer not to answer* was also included) was self-reported. Foreign background (as defined by Statistics Sweden; [20] see above) was based on the participants’ response to questions about their own and their parents’ country of birth.

Gender-stratified internal consistency scores for all other measures are found in Supplement A, Table A1 (Cronbach’s α ≥ 0.72, McDonald’s ω ≥ 0.80 in all instances).

*Non-suicidal self-injury* was measured using the 9-item version of the Deliberate Self-Harm Inventory (DSHI-9r) [21, 22]. Respondents were asked to rate, on a 7-point scale ranging from 0 (*never*) to 6 (*more than five times*), how often over the past 6 months they had engaged in each of nine forms of self-injury: (1) cutting their wrists, arms or other body areas; (2) minor cutting that causes bleeding; (3) burning with cigarette, lighter or match; (4) carving words, pictures, etc. into one’s skin; (5) severe scratching that causes bleeding; (6) biting oneself so as to break the skin; (7) sticking sharp objects into one’s skin; (8) punching oneself/banging one’s head, and (9) preventing wounds from healing. The total score (range 0–54) is the sum of the item scores. In accordance with previously established cut-offs and suggested definitions of clinically relevant NSSI [4, 16], we set the cut-off for *repetitive NSSI* as a total score of ≥ 5, while a total score of *≥* 1 is referred to as *any NSSI*.

*Disordered eating* was assessed using the 8-item Risk Behaviour related to Eating Disorders scale (RiBED-8) [23, 24]. As the RiBED-8 is a valid measure of DE among girls but not among boys [23], we analysed the results only for girls. The respondents rated how frequently they engaged in eight kinds of eating-related thoughts or behaviours on a 4-point scale from 1 (*almost never/never*) to 4 (*very often*), from which a total sum score was calculated. The RiBED-8 is strongly correlated (*r* = 0.76) with the Sick, Control, One, Fat, Food (SCOFF) questionnaire, a widely used screening tool for measuring DE among adolescents [25]. In addition to the RiBED-8 total score, we used a high-risk DE cut-off of critical responses (answering at/above a cutoff as defined for each item by Waaddegaard et al. [23]. ) on six or more items. This cut-off generated prevalence estimates in 2007/2008 that align with general prevalence estimates for eating disorders in Sweden [5]. 

*Mental health problems* were assessed using the Strengths and Difficulties Questionnaire – self-report version (SDQ-s) [26, 27]. Items were rated on a 3-point agreement scale (0 = *not true*, 2 = *certainly true*) based on the past six months. A total psychological difficulties score is calculated as the sum of scores on four subscales (5 items per subscale) targeting hyperactivity/inattention, emotional symptoms, conduct problems, and peer problems. In this study, we applied a clinically relevant cutoff of ≥ 18 (of a maximum of 40) for the total score [6]. 

## Procedure and ethics

Ethical approval was obtained from the Regional Ethics Committee at Lund University in 2006 (registration number 2006/49, for the 2007 and 2008 collections) and from the Swedish Ethical Review Authority in 2021 (registration number 2021-06695-01, for the 2024 and 2025 collections).

Data in both 2007/2008 and 2024/2025 was collected during the spring semester (February–April) in collaboration with municipal authorities and regular schools in the study area. Each school’s headmaster was contacted and consented to their school’s participation. Written information detailing the study objectives was sent to students and their parents. Students could opt out by informing their teachers, while parents could opt out on behalf of their child by contacting the teachers or researchers directly. All participating students provided consent; for those under age 15 years, passive parental consent was obtained, as approved by the Swedish Ethical Review Authority.

Data collection took place in each school during a designated lesson. Teachers were present but did not participate in administering the questionnaire (paper-pencil in 2007/2008, digitally in 2024/2025), which was done by research assistants from Lund University. A clinically trained psychologist was available on-site, by phone, or via email to address any potential concerns or adverse effects during or after data collection. Students were informed that participation was voluntary, with no need to provide a reason for opting out. Participants were identified using numeric codes to maintain confidentiality and to match data between waves.

### Statistical analysis

Analyses were either stratified by gender (girl or boy) or conducted with girls only (with regards to DE). Participants reporting *other/do not want to answer* in 2024/2025 were excluded from all analyses as this response option was unavailable in 2007/2008. The handling of missing data and statistical modelling of time trends are described in detail in Supplement B; the latter is summarized below. All analyses were run in R (version 4.5.1).

Research question 1, which examined the effects of period (2007/2008 vs. 2024/2025) on prevalence of NSSI, DE, and mental health problems, was assessed through mixed modelling while covarying for wave and foreign background. Research questions 2 and 3 were addressed with stepwise binominal generalized modelling, including the same covariates. For research question 2, we compared risk ratios between periods for model-estimated prevalences of each combination of scoring above/below clinically relevant cutoffs on the measures of NSSI/DE and mental health problems with Bonferroni-corrected *z*-tests. For research question 3, we considered two model terms: (i) *repetitive NSSI/high-risk DE×mental health problems* interaction at step 1 and (ii) *period×repetitive NSSI/high-risk DE×mental health problems* interaction at step 2, such that statistical significance of the latter would indicate a difference in the strength of the association (estimated by the former) across periods.

## Results

### Participants

During Period 1, approximately 90% of students responded (2007: 992 of 1064 eligible students in the class lists; 2008: 987 of 1098). In Period 2, response rates reached nearly 80% (2024: 810 of 1026; 2025: 836 of 1078). Reasons for non-response included absenteeism, switching school before data collection, and not wanting to participate.

Table 1 provides detailed demographics for the samples at each data collection point. As in the community population at large, the proportion of students with foreign background increased markedly between periods, highlighting background as a relevant covariate.

**Table 1:** Demographic data

	Period 1		Period 2	
	2007 (*N* = 992)	2008 (*N* = 987)	2024 (*N* = 810)	2025 (*N* = 836)
Age – *M* (*SD*)	13.71 (0.68)	14.76 (0.69)	13.87 (0.71)	14.79 (0.69)
Grade – *n* (%)				
Grade 7	507 (51.1)	-	431 (53.2)	-
Grade 8	485 (48.9)	512 (51.9)	379 (46.8)	444 (53.1)
Grade 9	-	475 (48.1)	-	392 (46.9)
Gender – *n* (%)				
Girls	500 (50.4)	504 (51.1)	376 (46.4)	384 (45.9)
Boys	492 (49.6)	483 (48.9)	419 (51.7)	426 (51.0)
Other/Do not want to answer	-	-	15 (1.9)	26 (3.1)
Background – *n* (%)				
Swedish	837 (84.4)	833 (84.4)	609 (75.2)	606 (72.5)
Foreign^a^	152 (15.3)	147 (14.9)	196 (24.2)	224 (26.8)
Missing data	3 (0.3)	7 (0.7)	5 (0.6)	6 (0.7)

”Other/Do not want to answer” was an available response option for gender only in 2024/2025

^a^ Operationalized as the participant being born abroad with at least one parent born abroad as well or being born in Sweden with both parents being born abroad

### Time trends in NSSI, DE, and mental health problems

Unadjusted prevalence rates are provided in Supplement A, Table A2. Adjusted time trends are summarized in Table 2, with details available in Supplement A, Tables A3–A5.

**Table 2:** Adjusted time trends between 2007/2008 and 2024/2025 in non-suicidal self-injury, disordered eating, and mental health problems.

	Girls – *2007/2008* (0) vs. *2024/2025* (1)			Boys – *2007/2008 *(0) vs. *2024/2025* (1)		
	*OR* [95% *CI*]	*b* [95% *CI*]	*p*	*OR* [95% *CI*]	*b* [95% *CI*]	*p*
Non-suicidal self-injury (NSSI)						
*Repetitive NSSI *(1) vs.* no repetitive NSSI* (0)	4.19 [2.37–7.41]		< 0.0001	0.72 [0.32–1.65]		0.4428
*Any NSSI* (1) vs.* no NSSI *(0)	1.21 [0.71–2.08]		0.4732	0.23 [0.09–0.56]		0.0014
Frequency of engagement^a^		0.70 [0.49–0.91]	< 0.0001		0.54 [-0.14–1.22]	0.1171
Disordered eating (DE)						
*High-risk DE* (1) vs. *no high-risk DE* (0)	2.90 [0.85–9.83]		0.0882			
Standardized mean score		0.17 [0.04–0.30]	0.0097			
Mental health problems						
*At/above clinically relevant threshold* (1) vs. *below threshold* (0)	7.50 [2.73–20.61]		0.0001	2.67 [0.97–7.35]		0.0578
Standardized mean score		0.69 [0.58–0.81]	< 0.0001		0.33 [0.22–0.43]	< 0.0001

All models included wave (i.e., *Baseline: 2007*,* 2024* or *Follow-up: 2008*,* 2025*) and student background (*Swedish* or *foreign*) as fixed effects and participant random intercepts. DE among boys is not statistically modelled due to the measure’s limited validity for this group. All estimates are provided in Supplement A (Table A3 − A5). *CI* Confidence interval, *OR* Odds ratio

^a^ Only calculated on participants reporting any NSSI (≥ 1 instances)

#### Time trends in NSSI

Among girls, the unadjusted prevalence of repetitive NSSI increased from 21.0%/25.3% in 2007/2008 to 39.7%/33.0% in 2024/2025 (*OR*_adjusted_ = 4.19, 95% *CI* = 2.37–7.41, *p* < 0.0001). However, the change in the prevalence of any NSSI (2007/2008: 41.5%/42.8%; 2024/2025: 39.7%/35.6%) was not statistically significant (*p* = 0.4732). In contrast, the increase in estimated count mean score on the DSHI-9r was statistically significant among girls who self-injured (*b*_adjusted_ = 0.70, 95% *CI* = 0.49–0.91, *p* < 0.0001).

Among boys, on the other hand, the unadjusted prevalence of repetitive NSSI (2007/2008: 15.8%/16.4%; 2024/2025: 14.9%/10.7%) and of any NSSI (2007/2008: 38.1%/36.7%; 2024/2025: 26.4%/22.5%) declined. Only the decline in any NSSI was statistically significant after adjustment (*OR*_adjusted_ = 0.23, 95% *CI* = 0.09–0.56, *p* < 0.0014). There was no change in count mean score on the DSHI-9r (*p* = 0.1171).

#### Time trends in DE

Among girls, the raw prevalence of high-risk DE more than doubled from 4.4%/6.8% in 2007/2008 to 11.6%/13.8% in 2024/2025, although this trend was not statistically significant (*p* = 0.0882). Girls also reported an increase in mean DE scores (*β*_adjusted_ = 0.17, 95% *CI* = 0.04–0.30, *p* = 0.0097).

#### Time trends in mental health problems

The proportion of girls with clinically relevant scores representing mental health problems increased from 10.3%/9.2% in 2007/2008 to 33.4%/29.5% in 2024/2025 (*OR*_adjusted_ = 7.50, 95% *CI* = 2.73–20.61, *p* = 0.0001). Their mean scores also increased (*β*_adjusted_ = 0.69, 95% *CI* = 0.58–0.81, *p* < 0.0001).

The proportion of boys passing the cut-off on mental health problems also increased between 2007/2008 and 2024/2025 (6.3%/8.8% to 16.7%/14.9%), though only at marginal statistical significance (*OR*_adjusted_ = 2.67, 95% *CI* = 0.97–7.35, *p* = 0.0578). The increase in boys’ mean was statistically significant, but to a lesser degree than that of girls (*β*_adjusted_ = 0.33, 95% *CI* = 0.22–0.43, *p* < 0.0001).

### Time trends in the prevalence of repetitive NSSI/high-risk DE in combination with clinically relevant mental health problems

Supplement A, Table A7 presents the unadjusted prevalence rates from 2007/2008 to 2024/2025 for repetitive NSSI and high-risk DE in conjunction with clinically relevant mental health problems: (1) below both thresholds, (2) at/above the behavioural measure only, (3) at/above the cutoff for clinically relevant mental health problems only, and (4) at/above both.

**Fig. 1:**
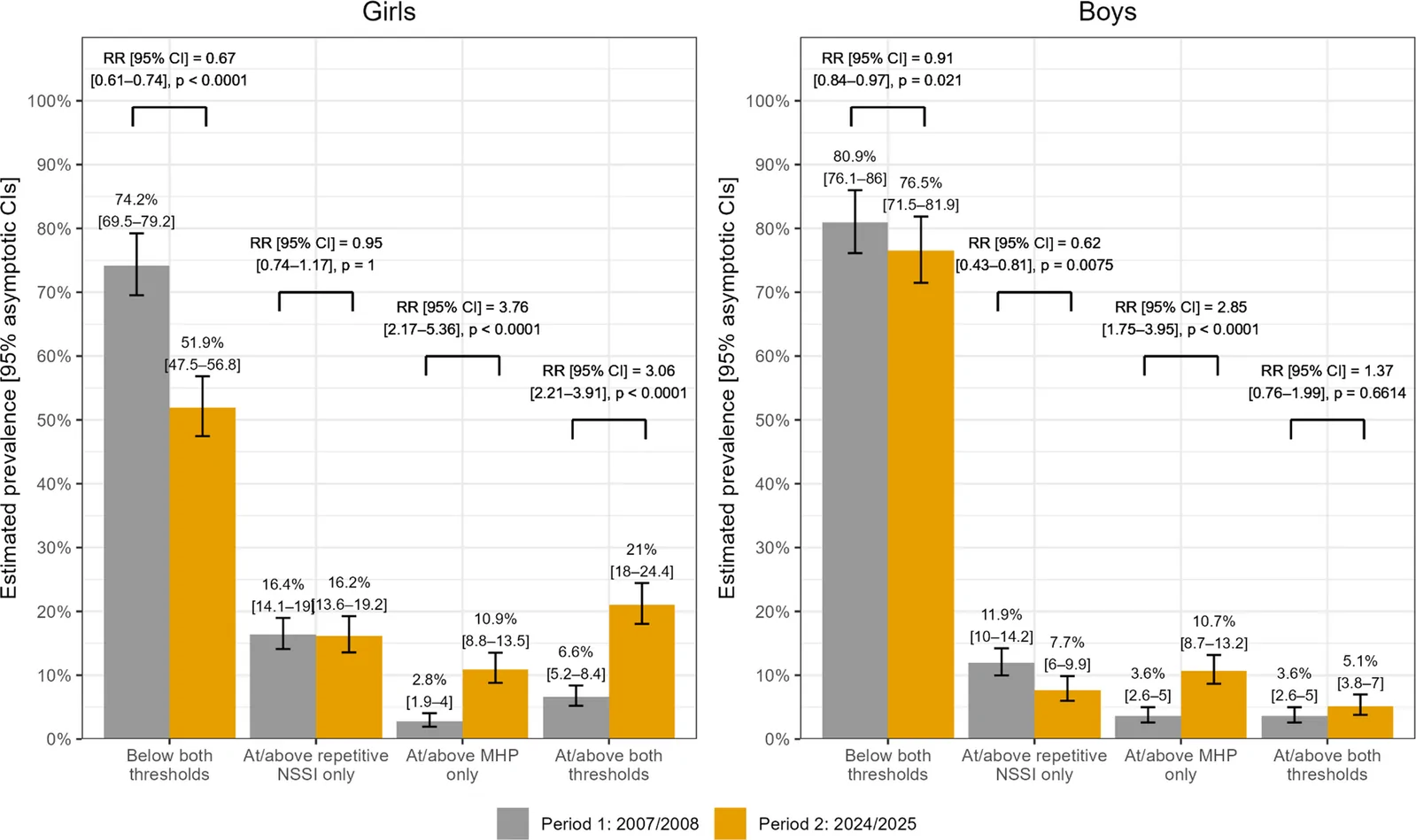
Time trends in proportion of adolescents scoring at/above or below thresholds for repetitive non-suicidal self-injury (NSSI) and clinically relevant mental health problems (MHP), adjusted for wave and foregin background. *p-*values are Bonferroni corrected (4 comparisons). Step 2 of Supplement A, Table A8, provides the model statistics used to estimate prevalence. *CI* = Confidence Interval; *RR* = Risk Ratio

Figure 1 illustrates that among girls, the adjusted prevalence of repetitive NSSI in combination with clinically relevant mental health problems increased from 6.6% to 21.0%; (*RR*_adjusted_ = 3.06, 95% *CI* = 2.20–5.42, *p*_bonferroni_ < 0.0001), whereas repetitive NSSI without mental health problems remained unchanged (*p*_bonferroni_ = 1.0000). In contrast, boys reported no statistically significant change in NSSI with mental health problems (*p*_bonferroni_ *=* 0.5289) and a statistically significant decrease in NSSI without mental health problems (*RR*_adjusted_ = 0.63, 95% *CI* = 0.44–0.82, *p*_bonferroni_ = 0.0100). The prevalence of mental health problems without repetitive NSSI increased among both girls and boys (*RR*_adjusted_ = 2.85–3.81, *p*_bonferroni_ ≤ 0.0001).

**Fig. 2:**
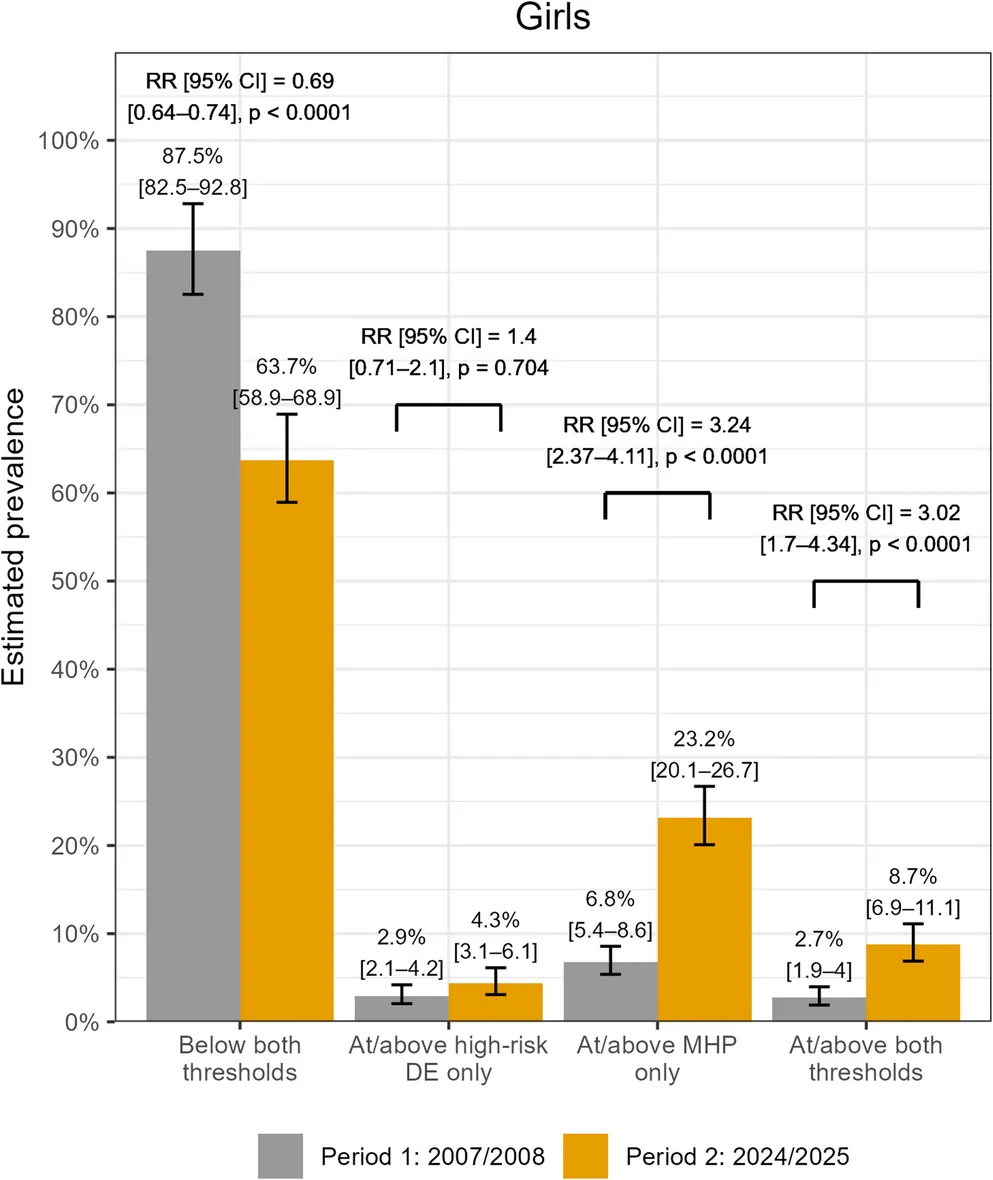
Time trends in proportion of adolescent girls scoring at/above or below thresholds for high-risk disordered eating (DE) and clinically relevant mental health problems (MHP), adjusted for wave and foregin background. *p-*values are Bonferroni corrected (4 comparisons). Step 2 of Supplement A, Table A9, provides the model statistics used to estimate prevalence. *CI* = Confidence Interval; *RR* = Risk Ratio

As seen in Fig. 2, there was a three-fold increase in the adjusted prevalence of high-risk DE combined with clinically relevant mental health problems among girls (*RR*_adjusted_ = 3.02, 95% *CI* = 1.70–4.34, *p*_bonferroni_ = 0.0001), whereas the prevalence of high-risk DE without mental health problems remained unchanged (*p*_bonferroni_ = 0.7040). There was also an increase in prevalence of mental health problems without high-risk DE among girls (*RR*_adjusted_ = 3.26, 95% *CI* = 2.39–4.13, *p*_bonferroni_ < 0.0001).

### Time trends in the interconnectivity of repetitive NSSI/high-risk DE with clinically relevant mental health problems

For girls, the findings in Supplement A, Table A8 support strong interconnectivity between repetitive NSSI and clinically relevant mental health problems (*RR*_adjusted_ = 8.12, 95% *CI* = 6.3–10.46; *p* < 0.0001), with a marginal statistically significant decrease in the strength of the association over time (*RR*_adjusted_ = 0.57, 95% *CI* = 0.33–1.01; *p* = 0.0525). There also was strong interconnectivity between high-risk DE and clinically relevant mental health problems among girls (*RR*_adjusted_ = 8.51, 95% *CI* = 6.05–11.97; *p* < 0.0001; Supplementary A, Table A9), which decreased over time (*RR*_adjusted_ = 0.46, 95% *CI* = 0.22–0.93; *p* = 0.0310).

For boys (Supplement A, Table A8) there was strong interconnectivity between repetitive NSSI and clinically relevant mental health problems (*RR*_adjusted_ = 5.03, 95% *CI* = 3.66–6.92, *p* < 0.0001), but this did not change over time (*RR*_adjusted_ = 0.71, 95% *CI* = 0.37–1.38, *p* = 0.3124).

## Discussion

This school-based study draws on self-report data from representative cohorts of Swedish adolescents collected in 2007/2008 and 2024/2025 (baseline and one-year follow-up of each cohort). It is the first study to examine secular gender-stratified trends in NSSI, DE, and their co-occurrence with other mental health problems, using validated multi-item scales with cut-offs for clinically relevant problems.

Consistent with previous research [1–3, 6], the present study found that general mental health problems have increased among both girls and boys over the past two decades, while NSSI increased only among girls. The unique contribution of the present study, however, is the findings for the time trends in the prevalence of NSSI and DE with versus without concomitant mental health problems.

The proportion of girls with repetitive NSSI combined with mental health problems increased about threefold (from around 7% to around 21%) from 2007/2008 to 2024/2025. In contrast, no increase was seen in the proportion of girls who engaged in repetitive NSSI without passing the cutoff for mental health problems. This strongly suggests that the increase in NSSI during the past two decades [6–8] reflects a trend towards worsening mental health among girls, rather than NSSI becoming increasingly normalized or detached from underlying distress.

Another interesting finding is that the proportion of girls who reported any NSSI did not increase. This suggests a kind of polarization, in the sense that the severity of problems among those who reported NSSI worsened during the 17-year period. A similar pattern was found by Sourander et al. [6], who reported that there was an increase specifically in the percentage of Finnish girls who engaged in NSSI multiple times. These findings indicate that a potential shift in the profiles of NSSI engagement, such that relatively fewer girls are now experimenting with NSSI on only a few occasions; instead, those who start may be more likely to continue and engage in it repetitively and with greater severity. This could have implications for early intervention: identifying and supporting girls who engage even in low-frequency NSSI might be critical to prevent escalation.

The findings on DE among girls show a similar pattern. The proportion of girls who engaged in high-risk DE combined with mental health problems also increased about threefold (from around 3% to around 9%). However, as for NSSI, there was no increase in the proportion of girls who engaged in high-risk DE without passing the cut-off for mental health problems, suggesting that what has increased are the clinically relevant forms of DE. This is consistent with other findings indicating rising trends in eating disorders among adolescent girls (e.g [28, 29]), . One limitation of the present study, however, is that we could not analyze possible trends in DE among boys, as the instrument we used does not cover the muscularity-oriented manifestations characteristic of DE among some boys [30]. 

Taken together, the findings are partly inconsistent with the hypothesis that the increase in self-reported mental health problems might be explained by prevalence inflation [19], at least among girls. If we are correct in arguing that self-reports of behaviours are less biased by trends of “pathologizing the normal” than are self-reports of inner symptoms, then it is unlikely that the threefold increase in the prevalence of mental problems in combination with NSSI or DE is attributable to such pathologization. On the other hand, because we observed even greater increases (i.e., close to fourfold) in the proportion of girls who passed the cut-off for mental health problems but did not show repetitive NSSI or high-risk DE, and the results indicated a trend towards a weakened interconnectivity between mental health problems and NSSI, part of the observed trends is still open to an interpretation in terms of prevalence inflation. Regardless, the findings point to a need for increased attention and action from the healthcare system with regard to girls’ mental health.

The results among the boys suggest a different picture, as we observed no increase in NSSI, neither per se nor in combination with mental health problems. On the contrary, the prevalence rate of any NSSI decreased among boys, and they also showed a non-significant trend towards a decrease in repetitive NSSI. There was a trend towards increasing mental health problems, however (the prevalence rate more than doubled), but this increase was only marginally statistically significant.

Regarding gender differences in the prevalence of NSSI, previous research has produced various findings. Gender differences tend to be larger in clinical samples than in community samples, with girls/women significantly outnumbering boys/men in clinical samples (e.g [31, 32]), . It has been unclear, however, how such findings should be interpreted. Are the results due to real differences in clinically relevant NSSI between genders, differences in treatment-seeking behavior, or a combination of both? The present findings suggest that real differences in clinically relevant NSSI are involved. As seen in Fig. 1, the combination of repetitive NSSI with clinically relevant mental health problems was considerably more frequent among girls than among boys both in 2007/2008 (6.6% vs. 3.6%) and in 2024/2025 (21% vs. 5.1%). If this combination of repetitive NSSI with clinically relevant mental health problems is considered a marker of clinically relevant NSSI, this suggests that the gender difference in clinically relevant NSSI has grown over the last few decades.

This may be contrasted with the prevalence of clinically relevant mental health problems *without* repetitive NSSI, as seen in Fig. 1, which showed no sign of gender differences in either 2007/2008 (2.8% vs. 3.6%) or 2024/2025 (10.9% vs. 10.7%). Importantly, this indicates a rather similar degree of deterioration in mental health among girls and boys, if we disregard NSSI. These results also suggest that boys’ psychological difficulties should not be underestimated – the nature of their psychological difficulties deserves particular study, such as to how these difficulties combine with other categories of suffering and self-destructive/harmful behaviors.

The present study has several strengths, including the secular, repeated design with two pairs of one-year interval assessments 17 years apart; the high response rate; and the representativeness of the studied municipality for Sweden in general. However, as shown in the Global Burden of Disease study [1], there are differences in prevalence rates and trends of various mental disorders between countries, meaning that the generalizability of this study is limited to regions with shared meso-, macro-, and chronosystems. The 17-year period between 2007/2008 and 2024/2025 was a period of immense societal change, such as the rise of social media/smartphones and the COVID-19 pandemic. However, the design of the present study does not make it possible to analyse the effects of such changes on the increase in mental health problems among adolescents. One social change that could be controlled for was the large immigration into Sweden during this period. Importantly, the percentage of students with a foreign background increased significantly from about 15% in 2007–2008 to about 25% in 2023–2024 (see Table 1), reflecting a general trend in Sweden due largely to a large wave of asylum seekers from Syria, Iraq, and Afghanistan. Because many of these immigrants had fled from war and unrest, it is possible that their mental health would differ from that of the general population in Sweden. Interestingly, however, the girls with foreign background showed significantly less NSSI than did other girls (see Supplement A, Table A3) and did not report more mental health problems (see Supplement A, Table A5). The increased mental health problems among girls, therefore, cannot be attributed to this demographic change.

In addition, estimates are aggregated at the population and scale level. Therefore, important demographic subgroups (e.g., in relation to background or socioeconomic status) and nuances in symptomology (e.g., what kind of mental health problems co-occurred with NSSI/DE) are not addressed and require further study. An additional limitation is that we did not have an instrument to measure DE as it often manifests in boys. The study is also limited by the lack of consideration of well-being aspects; it remains to be explained why rising rates of mental health problems have not been matched by comparable declines in life satisfaction [2, 3]. 

## Conclusion

The findings indicate a three-fold increase between 2007 and 2025 among girls in the prevalence of repetitive NSSI/high-risk DE when combined with other mental health problems, but no increase in the prevalence of NSSI/high-risk DE without co-occurring mental health problems. This strongly suggests that the rising trends in the prevalence of NSSI and DE among girls reflect a worsening of their mental health, rather than a normalization of NSSI/DE in certain subgroups of adolescents. No increasing trends in the prevalence rates of NSSI were seen among boys.

Overall, the increase over the past two decades concerns not NSSI and DE as stand-alone problems, but NSSI and DE as expressions of, and possible complications of, other mental health problems. These time trends, which appear to be specific for girls, require increased attention and action from the healthcare system. The increasing severity of problems among those who reported NSSI during the 17-year period also suggest that it might be increasingly important to identify and support girls who engage even in low-frequency NSSI to prevent escalation. It would also be important for further research to address potential factors that can explain these time trends as well as interventions that can protect against these developments at the individual level.

## Supplementary Information

Below is the link to the electronic supplementary material.

Supplementary Material A

Supplementary Material B

## Data Availability

Individual-level participant data cannot be openly shared due to its personal sensitive nature (Swedish ethics review act, 2003:460, 3 §). Individual-level participant data may be shared, in a de-identified form, after a project proposal has been approved by the Swedish Ethical Review Authority (Swedish ethics review act, 2003:460, 6 §) and signage of a data access agreement. Inquiries should be directed to DD (daiva.daukantaite@psy.lu.se).
