# Supplementary material for: Time trends in non-suicidal self-injury, disordered eating, and mental health problems between 2007 and 2025: a comparison of two Swedish adolescent cohorts
Source: Eur Child Adolesc Psychiatry. 2026 Jun 22;35(10):3325–34. doi: 10.1007/s00787-026-03105-4 (PMC13696555; doi:10.1007/s00787-026-03105-4)
Supplement: Supplementary file 1 [file 787_2026_3105_MOESM1_ESM.docx]

**Table A1.**

*Internal consistency estimates for all measures in each wave and period.*

| Variable (measure) | | Period 1: | | | | |  | Period 2: | | | | |
| --- | --- | --- | --- | --- | --- | --- | --- | --- | --- | --- | --- | --- |
|  |  | 2007 | |  | 2008 | |  | 2024 | |  | 2025 | |
|  |  | α | ω |  | α | ω |  | α | ω |  | α | ω |
| Non-suicidal self-injury (DSHI-9r) | |  |  |  |  |  |  |  |  |  |  |  |
|  | Girls | 0.92 | 0.94 |  | 0.90 | 0.93 |  | 0.90 | 0.92 |  | 0.89 | 0.92 |
|  | Boys | 0.88 | 0.91 |  | 0.93 | 0.95 |  | 0.95 | 0.96 |  | 0.91 | 0.93 |
| Disordered eating (RiBED-8) | |  |  |  |  |  |  |  |  |  |  |  |
|  | Girls | 0.80 | 0.83 |  | 0.83 | 0.87 |  | 0.89 | 0.91 |  | 0.90 | 0.92 |
| Total psychological difficulties (SDQ-s) | |  |  |  |  |  |  |  |  |  |  |  |
|  | Girls | 0.72 | 0.82 |  | 0.77 | 0.80 |  | 0.80 | 0.84 |  | 0.81 | 0.85 |
|  | Boys | 0.77 | 0.80 |  | 0.76 | 0.80 |  | 0.80 | 0.83 |  | 0.79 | 0.83 |

*Note*. DSHI-9r = Deliberate Self-Harm Inventory, 9-item version, revised; RiBED-8 = Risk Behaviour related to Eating Disorders; SDQ-s = Strengths and Difficulties questionnaire–Self-report version.

**Table A2.**

*Unadjusted prevalence rates and means of non-suicidal self-injury, disordered eating, and mental health problems.*

| Variable | | All participants | |  | Girls | |  | Boys | |
| --- | --- | --- | --- | --- | --- | --- | --- | --- | --- |
|  |  | Period 1: 2007/2008 | Period 2: 2024/2025 |  | Period 1: 2007/2008 | Period 2: 2024/2025 |  | Period 1: 2007/2008 | Period 2: 2024/2025 |
| **Non-suicidal self-injury (NSSI)** | | |  |  |  |  |  |  |  |
| Any NSSI – *n*, % [95% *CI*] | | |  |  |  |  |  |  |  |
|  | Baseline | 408, 41.5% [38.5–44.7] | 305, 39.7% [36.2–43.3] |  | 223, 45.0% [40.5–49.5] | 195, 53.4% [48.2–58.6] |  | 185, 38.1% [33.8–42.6] | 103, 26.4% [22.2–31.1] |
|  | Follow-up | 420, 42.8% [39.7–46.0] | 289, 35.6% [32.3–39.0] |  | 242, 48.6% [44.1–53.1] | 184, 48.9% [43.8–54.1] |  | 175, 36.7% [32.4–41.2] | 93, 22.5% [18.6–26.9] |
| Repetitive NSSI – *n*, % [95% *CI*] | | |  |  |  |  |  |  |  |
|  | Baseline | 181, 18.4% [16.1–21.0] | 208, 27.1% [24.0–30.4] |  | 104, 21.0% [17.5–24.9] | 145, 39.7% [34.7–45.0] |  | 77, 15.8% [12.8–19.5] | 58, 14.9% [11.6–18.9] |
|  | Follow-up | 207, 21.1% [18.6–23.8] | 178, 21.9% [19.2–25.0] |  | 126, 25.3% [21.6–29.4] | 124, 33.0% [28.3–38.0] |  | 78, 16.4% [13.2–20.1] | 44, 10.7% [7.9–14.1] |
| Mean – *M* [95% *CI*] | | |  |  |  |  |  |  |  |
|  | Baseline | 3.52 [3.00–4.03] | 5.32 [4.57–6.08] |  | 4.32 [3.49–5.14] | 8.11 [6.85–9.37] |  | 2.70 [2.10–3.29] | 2.66 [1.86–3.45] |
|  | Follow-up | 3.81 [3.25–4.37] | 4.57 [3.87–5.27] |  | 4.55 [3.75–5.35] | 6.89 [5.69–8.09] |  | 2.80 [2.09–3.51] | 2.07 [1.42–2.71] |
| Conditional mean – *M*_conditional_ [95% *CI*]^a^ | | | |  |  |  |  |  |  |
|  | Baseline | 8.46 [7.40–9.53] | 13.41 [11.91–14.9] |  | 9.61 [8.02–11.19] | 15.18 [13.33–17.03] |  | 7.09 [5.74–8.43] | 10.06 [7.55–12.57] |
|  | Follow-up | 8.90 [7.77–10.03] | 12.85 [11.29–14.42] |  | 9.36 [7.94–10.77] | 14.08 [12.10–16.05] |  | 7.63 [5.91–9.35] | 9.17 [6.82–11.52] |
| **Disordered eating (DE)** | | |  |  |  |  |  |  |  |
| High-risk DE – *n*, % [95% *CI*] | | |  |  |  |  |  |  |  |
|  | Baseline | *Not estimated* | |  | 22, 4.4% [2.9–6.7] | 43, 11.6% [8.6–15.4] |  | *Not estimated* | |
|  | Follow-up |  |  |  | 34, 6.8% [4.8–9.4] | 53, 13.8% [10.6–17.8] |  |  |  |
| Mean score – *M* [95% *CI*] | | |  |  |  |  |  |  |  |
|  | Baseline | *Not estimated* | |  | 14.33 [13.94–14.72] | 15.1 [14.52–15.69] |  | *Not estimated* | |
|  | Follow-up |  |  |  | 14.64 [14.23–15.06] | 15.25 [14.66–15.84] |  |  |  |
| **Total psychological difficulties** | | | |  |  |  |  |  |  |
| At/above clinically relevant cutoff – *n*, % [95% *CI*] | | | |  |  |  |  |  |  |
|  | Baseline | 82, 8.3% [6.7–10.3] | 198, 24.5% [21.6–27.7] |  | 51, 10.3% [7.8–13.4] | 125, 33.4% [28.7–38.5] |  | 31, 6.3% [4.4–9.0] | 70, 16.7% [13.4–20.8] |
|  | Follow-up | 92, 9.3% [7.6–11.4] | 188, 22.6% [19.8–25.6] |  | 46, 9.2% [6.9–12.1] | 113, 29.5% [25.0–34.4] |  | 42, 8.8% [6.5–11.8] | 63, 14.9% [11.7–18.7] |
| Mean score – *M* [95% *CI*] | | |  |  |  |  |  |  |  |
|  | Baseline | 10.12 [9.80–10.45] | 13.25 [12.83–13.68] |  | 10.38 [9.92–10.85] | 14.57 [13.94–15.21] |  | 9.86 [9.42–10.31] | 12.09 [11.53–12.65] |
|  | Follow-up | 10.55 [10.23–10.87] | 12.88 [12.48–13.29] |  | 10.88 [10.43–11.32] | 14.19 [13.59–14.79] |  | 10.11 [9.66–10.57] | 11.63 [11.1–12.16] |

*Note. N* may differ within assessments due to partial missingness. Estimates for DE for the total sample/among boys are not provided due to the measure’s limited validity for these groups. Nominal variables were operationalized as follows: NSSI (*any NSSI*: ≥1 instances in the past 6 months, *repetitive NSSI*: ≥5 instances in the past 6 months), DE (*high-risk DE*: ≥6 critical answers), psychological difficulties (total score ≥18). *CI* = Confidence Interval

^a^ Only calculated on participants reporting any NSSI (≥1 instances).

**Table A3.**

*Adjusted time trends in non-suicidal self-injury (NSSI).*

| Effect | **Logistic regression:**  *Repetitive NSSI* (1) vs. *No repetitive NSSI* (0) | | | | | | |
| --- | --- | --- | --- | --- | --- | --- | --- |
|  | Girls | | |  | Boys | | |
|  | *b* (*SE*) | *OR* [95% *CI*] | *p* |  | *b* (*SE*) | *OR* [95% *CI*] | *p* |
| Intercept | -2.39 (0.35) |  | <0.0001 |  | -7.09 (0.47) |  | <0.0001 |
| Wave (0 = *Baseline*; 1 = *Follow-up*) | 0.01 (0.15) | 1.01 [0.75–1.37] | 0.9281 |  | -0.35 (0.25) | 0.71 [0.43–1.16] | 0.1689 |
| Period (0 = *2007/2008*; 1 = *2024/2025*) | 1.43 (0.29) | 4.19 [2.37–7.41] | <0.0001 |  | -0.32 (0.42) | 0.72 [0.32–1.65] | 0.4428 |
| Background (0 = *Swedish*; 1 = *Foregin*) | -1.18 (0.33) | 0.31 [0.16–0.59] | 0.0003 |  | 0.34 (0.50) | 1.40 [0.53–3.73] | 0.4965 |
| Random intercept (participant) | *σ*^2^ = 7.09 | | |  | *σ*^2^ = 72.84 | | |
|  | **Zero-inflated Poisson mixed modelling:** | | | | | | |
| Random effect correlation | -0.51 | | |  | 0.23 | | |
|  | Zero-inflated component –  *No NSSI* (1) vs. *Any NSSI* (0) | | | | | | |
|  | *α* (*SE*) | *OR* [95% *CI*] | *p* |  | *α* (*SE*) | *OR* [95% *CI*] | *p* |
| Intercept | -0.34 (0.22) |  | 0.1179 |  | -0.18 (0.29) |  | 0.5378 |
| Wave (0 = *Baseline*; 1 = *Follow-up*) | -0.10 (0.17) | 0.9 [0.65–1.26] | 0.5542 |  | 0.26 (0.17) | 1.30 [0.94–1.81] | 0.1174 |
| Period (0 = *2007/2008*; 1 = *2024/2025*) | -0.2 (0.27) | 0.82 [0.48–1.41] | 0.4732 |  | 1.48 (0.46) | 4.38 [1.77–10.82] | 0.0014 |
| Background (0 = *Swedish*; 1 = *Foregin*) | 1.20 (0.36) | 3.33 [1.66–6.68] | 0.0007 |  | -0.68 (0.66) | 0.51 [0.14–1.86] | 0.3064 |
| Random intercept (participant) | *σ*^2^ = 9.27 | | |  | *σ*^2^ = 2.51 | | |
|  | Poisson regression component –  only computed on participants reporting ≥1 instances | | | | | | |
|  | *b* (*SE*) | [*b* 95% *CI*] | *p* |  | *b* (*SE*) | [*b* 95% *CI*] | *p* |
| Intercept | 1.20 (0.11) | [0.98–1.41] | <0.0001 |  | 0.95 (0.34) | [0.29–1.61] | 0.0050 |
| Wave (0 = *Baseline*; 1 = *Follow-up*) | -0.05 (0.02) | [-0.09–0.00] | 0.0514 |  | 0.04 (0.04) | [-0.05–0.12] | 0.4170 |
| Period (0 = *2007/2008*; 1 = *2024/2025*) | 0.70 (0.11) | [0.49–0.91] | <0.0001 |  | 0.54 (0.35) | [-0.14–1.22] | 0.1171 |
| Background (0 = *Swedish*; 1 = *Foregin*) | -0.29 (0.14) | [-0.57–-0.02] | 0.0380 |  | -0.71 (0.32) | [-1.34–-0.08] | 0.0271 |
| Random intercept (participant) | *σ*^2^ = 1.42 | | |  | *σ*^2^ = 3.93 | | |

*Note*. In the manuscript (Table 2), the coefficients for the zero-inflated component has been reversed to facilitate interpretation. Nominal outcomes were operationalized as follows: *no NSSI* (0 instances in the past 6 months)*, any NSSI* (≥1 instance in the past 6 months), *repetitive NSSI* (≥5 instances in the past 6 months). *CI* = Confidence Interval; *OR* = Odds Ratio; *SE* = Standard Error.

**Table A4.**

*Adjusted time trends in disordered eating (DE) among girls.*

| Effect | **Logistic mixed modelling:**  *High-risk DE* (1: ≥6 critical answers) vs. *below high-risk DE cutoff* (0: <6 critical answers) | | |  | **Linear mixed modelling** | | |
| --- | --- | --- | --- | --- | --- | --- | --- |
|  | *b* (*SE*) | *OR* [95% *CI*] | *p* |  | *β* (*SE*) | *β* [95% *CI*] | *p* |
| Intercept | -10.33 (0.74) |  | <0.0001 |  | 0.24 (0.05) | 0.24 [0.15–0.33] | <0.0001 |
| Wave (0 = *Baseline*; 1 = *Follow-up*) | 1.30 (0.40) | 3.69 [1.69–8.06] | 0.0011 |  | 0.05 (0.03) | 0.05 [-0.01–0.11] | 0.0805 |
| Period (0 = *2007/2008*; 1 = *2024/2025*) | 1.06 (0.62) | 2.90 [0.85–9.83] | 0.0882 |  | 0.17 (0.07) | 0.17 [0.04–0.30] | 0.0097 |
| Background (0 = *Swedish*; 1 = *Foregin*) | -0.45 (0.82) | 0.64 [0.13–3.20] | 0.5865 |  | -0.18 (0.08) | -0.18 [-0.34–  -0.02] | 0.0305 |
| Random intercept (participant) | *σ*^2^ = 131.60 | | |  | *σ*^2^ = 0.82 | | |
| Residual |  | | |  | *σ*^2^ = 0.36 | | |

*Note*. Due to the poor psychometric properties of the measure, trends in high-risk DE were not statistically modelled among boys. *CI* = Confidence Interval; *OR* = Odds Ratio; *SE* = Standard Error.

**Table A5.**

*Adjusted time trends in mental health problems.*

| Effect | **Logistic mixed modelling:**  *At/above cutoff* (1: total score ≥18) vs*. below cutoff* (0: total score <18) | | | | | | |
| --- | --- | --- | --- | --- | --- | --- | --- |
|  | Girls | | |  | Boys | | |
|  | *b* (*SE*) | *OR* [95% *CI*] | *p* |  | *b* (*SE*) | *OR* [95% *CI*] | *p* |
| Intercept | -8.84 (0.54) |  | <0.0001 |  | -8.99 (0.58) |  | <0.0001 |
| Wave (0 = *Baseline*; 1 = *Follow-up*) | -0.51 (0.28) | 0.60 [0.35–1.05] | 0.0720 |  | 0.18 (0.30) | 1.19 [0.66–2.16] | 0.5630 |
| Period (0 = *2007/2008*; 1 = *2024/2025*) | 2.01 (0.52) | 7.50 [2.73–20.61] | 0.0001 |  | 0.98 (0.52) | 2.67 [0.97–7.35] | 0.0578 |
| Background (0 = *Swedish*; 1 = *Foregin*) | -0.71 (0.64) | 0.49 [0.14–1.73] | 0.2694 |  | 0.16 (0.57) | 1.18 [0.38–3.62] | 0.7789 |
| Random intercept (participant) | *σ*^2^ = 148.12 | | |  | *σ*^2^ = 108.95 | | |
|  | **Linear mixed modelling** | | | | | | |
|  | *β* (*SE*) | *β* [95% *CI*] | *p* |  | *β* (*SE*) | *β* [95% *CI*] | *p* |
| Intercept | -0.12 (0.04) | -0.12 [-0.21–  -0.04] | 0.0039 |  | -0.26 (0.04) | -0.26 [-0.34–  -0.18] | <0.0001 |
| Wave (0 = *Baseline*; 1 = *Follow-up*) | 0.01 (0.03) | 0.01 [-0.04–0.07] | 0.5846 |  | -0.01 (0.03) | -0.01 [-0.07–0.05] | 0.7307 |
| Period (0 = *2007/2008*; 1 = *2024/2025*) | 0.69 (0.06) | 0.69 [0.58–0.81] | <0.0001 |  | 0.33 (0.05) | 0.33 [0.22–0.43] | <0.0001 |
| Background (0 = *Swedish*; 1 = *Foregin*) | -0.24 (0.07) | -0.24 [-0.38–  -0.09] | 0.0016 |  | 0.02 (0.06) | 0.02 [-0.11–0.15] | 0.7536 |
| Random intercept (participant) | *σ*^2^ = 0.69 | | |  | *σ*^2^ = 0.51 | | |
| Residual | *σ^2^ = 0.27* | | |  | *σ*^2^ = 0.36 | | |

*Note*. *CI* = Confidence Interval; *OR* = Odds Ratio; *SE* = Standard Error.

**Table A6.**

*Unadjusted prevalence rates of repetitive NSSI in combination with clinically relevant levels of mental health problems.*

| *n*, % [95% *CI*] | | All participants | |  | Girls | |  | Boys | |
| --- | --- | --- | --- | --- | --- | --- | --- | --- | --- |
|  |  | Period 1: 2007/2008 | Period 2: 2024/2025 |  | Period 1: 2007/2008 | Period 2: 2024/2025 |  | Period 1: 2007/2008 | Period 2: 2024/2025 |
| Below both thresholds | | |  |  |  |  |  |  |  |
|  | Baseline | 769, 78.5% [75.8–81.1] | 483, 62.9% [59.4–66.3] |  | 378, 76.4% [72.3–80.0] | 184, 50.4% [45.2–55.6] |  | 391, 80.8% [76.9–84.1] | 292, 74.9% [70.2–79.0] |
|  | Follow-up | 738, 75.3% [72.5–78.0] | 537, 66.4% [63.0–69.6] |  | 356, 71.6% [67.4–75.5] | 208, 55.5% [50.3–60.5] |  | 380, 79.7% [75.7–83.1] | 323, 78.6% [74.2–82.4] |
| At/above repetitive NSSI threshold only | | |  |  |  |  |  |  |  |
|  | Baseline | 131, 13.4% [11.3–15.7] | 96, 12.5% [10.3–15.1] |  | 68, 13.7% [10.9–17.2] | 59, 16.2% [12.6–20.4] |  | 63, 13.0% [10.2–16.4] | 34, 8.7% [6.2–12.1] |
|  | Follow-up | 150, 15.3% [13.1–17.7] | 88, 10.9% [8.9–13.3] |  | 95, 19.1% [15.8–22.9] | 56, 14.9% [11.6–19.0] |  | 55, 11.5% [8.9–14.8] | 27, 6.6% [4.5–9.5] |
| At/above MPH threshold only | | | |  |  |  |  |  |  |
|  | Baseline | 31, 3.2% [2.2–4.5] | 77, 10.0% [8.0–12.4] |  | 14, 2.8% [1.6–4.8] | 36, 9.9% [7.1–13.5] |  | 17, 3.5% [2.1–5.7] | 40, 10.3% [7.5–13.8] |
|  | Follow-up | 35, 3.6% [2.5–5.0] | 95, 11.7% [9.6–14.2] |  | 15, 3.0% [1.8–5.0] | 44, 11.7% [8.7–15.5] |  | 19, 4.0% [2.5–6.3] | 44, 10.7% [8.0–14.2] |
| At/above both thresholds | | |  |  |  |  |  |  |  |
|  | Baseline | 48, 4.9% [3.7–6.5] | 112, 14.6% [12.2–17.3] |  | 35, 7.1% [5.0–9.8] | 86, 23.6% [19.4–28.3] |  | 13, 2.7% [1.5–4.7] | 24, 6.2% [4.1–9.1] |
|  | Follow-up | 57, 5.8% [4.5–7.5] | 89, 11.0% [9.0–13.4] |  | 31, 6.2% [4.3–8.8] | 67, 17.9% [14.2–22.2] |  | 23, 4.8% [3.1–7.3] | 17, 4.1% [2.5–6.7] |

*Note*. *N* may differ within assessments due to partial missingness. Thresholds were operationalized as follows: *repetitive NSSI* (0−4 instances *vs*. ≥5 instances), *MHP* (total score 0−17 *vs*. total score ≥18). *CI* = Confidence Interval; NSSI = Non-Suicidal Self-injury; MHP = Mental health problems (SDQ-s psychological difficulties, total score).

**Table A7.**

*Unadjusted prevalence rates of high-risk DE in combination with clinically relevant levels of mental health problems among girls.*

| *n*, % [95% *CI*] | | Girls | |
| --- | --- | --- | --- |
|  |  | Period 1: 2007/2008 | Period 2: 2024/2025 |
| Below both thresholds | |  |  |
|  | Baseline | 433, 87.3% [84.0–90.0] | 234, 62.9% [57.7–67.8] |
|  | Follow-up | 438, 87.4% [84.1–90.1] | 251, 65.7% [60.7–70.4] |
| At/above high-risk DE threshold only | | |  |
|  | Baseline | 13, 2.6% [1.5–4.6] | 13, 3.5% [2.0–6.1] |
|  | Follow-up | 17, 3.4% [2.1–5.5] | 19, 5.0% [3.1–7.8] |
| At/above MHP threshold only | |  |  |
|  | Baseline | 41, 8.3% [6.1–11.1] | 95, 25.5% [21.2–30.3] |
|  | Follow-up | 29, 5.8% [4.0–8.3] | 78, 20.4% [16.6–24.9] |
| At/above both thresholds | |  |  |
|  | Baseline | 9, 1.8% [0.9–3.5] | 30, 8.1% [5.6–11.4] |
|  | Follow-up | 17, 3.4% [2.1–5.5] | 34, 8.9% [6.3–12.3] |

*Note*. *N* may differ within assessments due to partial missingness. Estimates for the total sample/among boys are not provided due to the DE measure’s limited validity for these groups. Thresholds were operationalized as follows: *high-risk DE* (0−5 critical answers *vs*. ≥6 critical answers), *MHP* (total score 0−17 *vs*. total score ≥18). *CI* = Confidence Interval, DE = Disordered Eating; MHP = Mental health problems (SDQ-s psychological difficulties, total score).

**Table A8.**

*Adjusted time trends in the interconnectivity between repetitive NSSI and clinically relevant levels of mental health problems, estimated with binominal generalized modelling.*

| Independent variable | | Girls | | |  | Boys | | |
| --- | --- | --- | --- | --- | --- | --- | --- | --- |
|  |  | *b* (*SE*) | *RR* [95% *CI*] | *p* |  | *b* (*SE*) | *RR* [95% *CI*] | *p* |
| **Step 1** | |  |  |  |  |  |  |  |
| Intercept | | -0.57 (0.03) |  | <0.0001 |  | -0.42 (0.03) |  | <0.0001 |
| Main terms | |  |  |  |  |  |  |  |
|  | Background (0 = *Swedish;* 1 = *Foregin*) | -1.47 (0.06) | 0.23 [0.21–0.26] | <0.0001 |  | -1.41 (0.05) | 0.24 [0.22–0.27] | <0.0001 |
|  | Wave (0 = *Baseline*; 1 = *Follow-up*) | -0.01 (0.04) | 0.99 [0.92–1.07] | 0.7875 |  | 0 (0.03) | 1 [0.93–1.06] | 0.8937 |
|  | Period (0 = *2007/2008*; 1 = *2024/2025*) | -0.15 (0.04) | 0.86 [0.79–0.92] | 0.0001 |  | -0.07 (0.03) | 0.93 [0.87–0.99] | 0.0294 |
|  | Repetitive NSSI (0 = *No repetitive NSSI;* 1 = *Repetitive NSSI*) | -1.39 (0.06) | 0.25 [0.22–0.28] | <0.0001 |  | -2.06 (0.07) | 0.13 [0.11–0.15] | <0.0001 |
|  | MHP (0 *= Below threshold*; 1 *= At/above threshold*) | -2.35 (0.1) | 0.1 [0.08–0.12] | <0.0001 |  | -2.46 (0.09) | 0.09 [0.07–0.1] | <0.0001 |
| Interaction terms | |  |  |  |  |  |  |  |
|  | NSSI×MHP | 2.09 (0.13) | 8.12 [6.3–10.46] | <0.0001 |  | 1.62 (0.16) | 5.03 [3.66–6.92] | <0.0001 |
| **Step 2** | |  |  |  |  |  |  |  |
| Intercept | | -0.48 (0.03) |  | <0.0001 |  | -0.41 (0.03) |  | <0.0001 |
| Main terms | |  |  |  |  |  |  |  |
|  | Background (0 = *Swedish;* 1 = *Foregin*) | -1.47 (0.06) | 0.23 [0.21–0.26] | <0.0001 |  | -1.41 (0.05) | 0.24 [0.22–0.27] | <0.0001 |
|  | Wave (0 = *Baseline*; 1 = *Follow-up*) | -0.02 (0.04) | 0.98 [0.91–1.06] | 0.6278 |  | 0 (0.03) | 1 [0.93–1.06] | 0.8886 |
|  | Period (0 = *2007/2008*; 1 = *2024/2025*) | -0.39 (0.05) | 0.68 [0.61–0.74] | <0.0001 |  | -0.1 (0.04) | 0.9 [0.84–0.97] | 0.0042 |
|  | Repetitive NSSI (0 = *No repetitive NSSI;* 1 = *Repetitive NSSI*) | -1.51 (0.08) | 0.22 [0.19–0.26] | <0.0001 |  | -1.92 (0.09) | 0.15 [0.12–0.18] | <0.0001 |
|  | MHP (0 *= Below threshold*; 1 *= At/above threshold*) | -3.28 (0.19) | 0.04 [0.03–0.05] | <0.0001 |  | -3.11 (0.17) | 0.04 [0.03–0.06] | <0.0001 |
| Interaction terms | |  |  |  |  |  |  |  |
|  | Repetitive NSSI×MHP | 2.37 (0.24) | 10.74 [6.77–17.02] | <0.0001 |  | 1.91 (0.25) | 6.79 [4.13–11.14] | <0.0001 |
|  | Period×Repetitive NSSI | 0.34 (0.12) | 1.4 [1.1–1.78] | 0.0067 |  | -0.36 (0.16) | 0.7 [0.51–0.95] | 0.0210 |
|  | Period×MHP | 1.73 (0.22) | 5.65 [3.66–8.71] | <0.0001 |  | 1.15 (0.20) | 3.15 [2.13–4.67] | <0.0001 |
|  | Period×Repetitive NSSI×MHP | -0.55 (0.29) | 0.57 [0.33–1.01] | 0.0525 |  | -0.34 (0.34) | 0.71 [0.37–1.38] | 0.3124 |

*Note*. The thresholds were operationalized as follows: *repetitive* *NSSI* (0−4 instances *vs*. ≥5 instances), *MHP* (total score 0−17 *vs*. total score ≥18). *CI* = Confidence Interval; MHP = Mental health problems (SDQ-s psychological difficulties, total score); NSSI = Non-suicidal self-injury. *RR* = Risk Ratio; *SE* = Standard Error.

**Table A9.**

*Adjusted time trends in the interconnectivity between high-risk DE and clinically relevant levels of mental health problems among girls, estimated with binominal generalized modelling.*

| Independent variable | | Girls: Step 1 | | |  | Girls: Step 2 | | |
| --- | --- | --- | --- | --- | --- | --- | --- | --- |
|  |  | *b* (*SE*) | *RR* [95% *CI*] | *p* |  | *b* (*SE*) | *RR* [95% *CI*] | *p* |
| Intercept | | -0.37 (0.03) | 0.69 [0.66–0.73] | <0.0001 |  | -0.32 (0.02) |  | <0.0001 |
| Main terms | |  |  |  |  |  |  |  |
|  | Background (0 = *Swedish;* 1 = *Foregin*) | -1.48 (0.05) | 0.23 [0.21–0.25] | <0.0001 |  | -1.47 (0.05) | 0.23 [0.21–0.26] | <0.0001 |
|  | Wave (0 = *Baseline*; 1 = *Follow-up*) | 0.01  (0.03) | 1.01 [0.94–1.07] | 0.8671 |  | 0.01 (0.03) | 1.01 [0.95–1.07] | 0.8709 |
|  | Period (0 = *2007/2008*; 1 = *2024/2025*) | -0.20 (0.03) | 0.82 [0.76–0.87] | <0.0001 |  | -0.37 (0.04) | 0.69 [0.64–0.74] | <0.0001 |
|  | High-risk DE (0 = *No high-risk DE;* 1 = *High-risk DE*) | -3.10 (0.13) | 0.05 [0.04–0.06] | <0.0001 |  | -3.39 (0.18) | 0.03 [0.02–0.05] | <0.0001 |
|  | MHP (0 *= Below threshold*; 1 *= At/above threshold*) | -1.75 (0.06) | 0.17 [0.15–0.2] | <0.0001 |  | -2.56 (0.12) | 0.08 [0.06–0.10] | <0.0001 |
| Interaction terms | |  |  |  |  |  |  |  |
|  | High-risk DE×MHP | 2.14  (0.17) | 8.51 [6.05–11.97] | <0.0001 |  | 2.49 (0.29) | 12.05 [6.88–21.09] | <0.0001 |
|  | Period×High-risk DE |  |  |  |  | 0.71 (0.25) | 2.03 [1.24–3.35] | 0.0052 |
|  | Period×MHP |  |  |  |  | 1.55 (0.14) | 4.72 [3.58–6.24] | <0.0001 |
|  | Period×High-risk DE×MHP |  |  |  |  | -0.79 (0.36) | 0.46 [0.22–0.93] | 0.0310 |

*Note*. Due to the poor psychometric properties of the measure, trends in high-risk DE were not statistically modelled among boys. The thresholds were operationalized as follows: *high-risk DE* (0−5 critical answers *vs*. ≥6 critical answers), *MHP* (total score 0−17 *vs*. total score ≥18). *CI* = Confidence Interval; DE = Disordered Eating; MHP = Mental health problems (SDQ-s psychological difficulties, total score); *RR* = Risk Ratio; *SE* = Standard Error.
