# Supplementary material for: Time trends in non-suicidal self-injury, disordered eating, and mental health problems between 2007 and 2025: a comparison of two Swedish adolescent cohorts
Source: Eur Child Adolesc Psychiatry. 2026 Jun 22;35(10):3325–34. doi: 10.1007/s00787-026-03105-4 (PMC13696555; doi:10.1007/s00787-026-03105-4)
Supplement: Supplementary file 2 [file 787_2026_3105_MOESM2_ESM.docx]

**Statistical analysis**

All analyses described below were run in R (version 4.5.1).

**Missingness**

Little’s^1^ missing completely at random (MCAR) test suggested that within each period and wave, missingness was not MCAR for girls/boys (Table B1: *p* ≤ 0.0569). Nevertheless, as the χ^2^-to-*df* ratio was <2, deviations from the MCAR assumption were interpreted as minor.^2^ Visual inspection (with *md.pattern* from *mice*, version 3.180)^3^ did not identify any discernible or prominent patterns, except for missingness on an entire scale (e.g., not answering any question on the Deliberate Self-Harm Inventory [DSHI-9r]). This was likely because the respondent skipped a page in the questionnaire.

**Table B1.**

*Overview of missingness in the measures.*

| Sample | | Partially incomplete cases | Number of unique patterns | Most cases within the same pattern | Most cases missing the same item | Little’s missing completely at random test | | | χ^2^-to-*df* ratio |
| --- | --- | --- | --- | --- | --- | --- | --- | --- | --- |
|  |  |  |  |  |  | χ^2^ | *df* | *p* |  |
| 2007 | |  |  |  |  |  |  |  |  |
|  | Girls | 47 | 31 | 5 | 10 | 1793.06 | 994 | < 0.0001 | 1.80 |
|  | Boys | 41 | 27 | 8 | 4 | 855.39 | 672 | < 0.0001 | 1.27 |
| 2008 | |  |  |  |  |  |  |  |  |
|  | Girls | 68 | 48 | 5 | 11 | 1702.77 | 1612 | 0.0569 | 1.06 |
|  | Boys | 72 | 28 | 4 | 5 | 1066.35 | 734 | < 0.0001 | 1.45 |
| 2024 | |  |  |  |  |  |  |  |  |
|  | Girls | 90 | 57 | 22 | 7 | 2041.39 | 1919 | 0.0258 | 1.06 |
|  | Boys | 118 | 53 | 32 | 52^a^ | 1560.06 | 1280 | < 0.0001 | 1.22 |
| 2025 | |  |  |  |  |  |  |  |  |
|  | Girls | 88 | 47 | 24 | 10 | 1690.80 | 1571 | 0.0180 | 1.08 |
|  | Boys | 90 | 44 | 28 | 14 | 1222.62 | 1078 | 0.0014 | 1.13 |

*Note*. Tested measures for girls included items from the Deliberate Self-Harm Inventory (DSHI-9r), Risk Behaviour related to Eating Disorders scale (RiBED-9), and Strengths and Difficulties Questionnaire – self-report version (SDQ-s), and among boys included items from the DSHI-9r and SDQ-s.

^a^ These values represent missingness in the DHSI-9r item assessing preventing wounds from healing; attributed to not answering any item in the DSHI-9r at all (*n* = 41) or random patterns (*n* = 11).

We opted to impute missing values when ≥50% of the scale items had valid responses, using the other items of that scale as predictors. Imputation was performed using predictive mean matching, which is suitable when the missing variable is not normally distributed.^4^ Across periods and waves, DSHI-9r item values were imputed in 125 instances (82.4% imputations comprised a single item within that scale), Risk Behaviour related to Eating Disorders scale (RiBED-8) values in 66 instances (girls only; 84.6% comprised a single item), and Strengths and Difficulties Questionnaire – self-report version (SDQ-s) values in 258 instances (81.8% comprised a single item). For cases where missingness exceeded 50% within a particular scale, that scale was treated as case-wise missing because there was not sufficient data for reliable imputation.

**Research question 1: Separate time trends in prevalence**

Time trends in each separate disturbance were assessed through three types of mixed models applied to a long dataset (where each observation is represented by one row): logistic models for dichotomized outcomes (below vs. at/above a cutoff); restricted maximum-likelihood linear models for disordered eating (DE) and total psychological difficulties (herein referenced as Mental Health Problems; MHP) standardized mean scores; and zero-inflated Poisson models (hybrid expectation-maximization/quasi-Newton optimization) for non-suicidal self-injury (NSSI) scores (further detail provided below). The logistic and linear models were fitted with packages *lme4* (version 1.1-37)^5^ and *afex* (version 1.5-0)^6^ in R.

$\mathrm{logit}(p_{ij})=\gamma_{00}+\gamma_{01}{(period}_{i})+b_{1}({wave}_{ij})+\gamma_{02}{(background}_{i})+u_{0i}$	(1)

$Y_{ij}=\gamma_{00}+\gamma_{01}{(period}_{i})+b_{1}({wave}_{ij})+\gamma_{02}{(background}_{i})+u_{0i}+\varepsilon_{ij}$	(2)

Equation 1 presents the logistic mixed model ($Y_{ij}\sim Bernoulli\left( p_{ij} \right)$) and equation 2 the linear mixed model, such that the outcome is modelled for individual *i* at measurement *j*, with notation *b* indicating level-1 variables and γ level-2 variables.^7^

In accordance with the study aims, the focus of the analysis across all mixed models was the nominal fixed effect of period of assessment (“period”: *2007/2008* vs*. 2024/2025*). In the logistic models, the effect was interpreted through the exponential of the “period” estimate, also known as the odds ratio.

Additionally, all models included the covariates “wave” (*Baseline: 2007, 2024* vs. *Follow-up: 2008, 2025*) and “background” (*Swedish* vs. *Foreign*). The latter accounts for changing proportions of participants with a foreign background, while the former adjusts the effect of period to reflect the average between waves. Ideally, “wave” should be modelled as a random slope to account for how individuals are expected to change at different rates;^7^ however, few assessments limited “wave” to a fixed effect. Nevertheless, each model included participant ID as a random intercept (*u*), to account for that most but not all participants responded twice (e.g., in 2007 *and* 2008).

***Zero-inflated Poisson mixed models.*** A considerable floor effect was suggested for the NSSI scores (skew_girls_ = 2.42, skew_boys_ = 4.54, across period and waves), which was due to excess zeroes (i.e., most do not self-injure). Therefore, we opted for a zero-inflated Poisson mixed model rather than a linear model. This mixed model has two components.^8^ The zero-inflated component is akin to logistic modelling, predicting the likelihood that the outcome does not occur (i.e., the likelihood of not self-injuring) ($Y_{ij}\sim Bernoulli\left( \pi_{ij} \right)$; equation 3). In turn, the Poisson component models the frequency of the outcome after excluding structural zeros (i.e., the frequency of engagement in NSSI after zero-scores in the DSHI-9r had been omitted) (equation 4). Meanwhile, in contrast to running separate or hurdle models, the zero-inflated Poisson model enables the random intercepts of each part to covary ($Cov(u_{0i}, v_{0i})$), capturing shared individual heterogeneity, which may impact both parts. The zero-inflated Poisson model was fitted with *GLMMadaptive* (version 0.9-7).^9^

$\mathrm{logit}(\pi_{ij})=\gamma_{00}^{(\pi)}+\gamma_{01}^{(\pi)}{(period}_{i})+b_{1}({wave}_{ij})+\gamma_{02}^{(\pi)}{(background}_{i})+u_{0i}$	(3)

$\log(\mu_{ij})=\gamma_{00}^{(\mu)}+\gamma_{01}^{(\mu)}{(period}_{i})+b_{1}({wave}_{ij})+\gamma_{02}^{(\mu)}{(background}_{i})+v_{0i}$	(4)

**Research question 2 and 3**

For research questions 2 and 3, we shifted from statistical modelling of individual-level risk to modelling group-level risk. This was motivated by the low intercepts of the mixed models, indicating rare outcomes (especially among boys) and consequent problems with estimating reliable proportions from even more complex mixed models. Therefore, we aggregated data within each period and wave, calculating the frequency (*n*) of each possible combination of the predictors (e.g., *n* participants with a foreign background scoring above the clinically relevant threshold for MHP and each behavioural indicator in the first wave in the first period). These aggregations were gender-stratified and performed separately for the two behavioural indicators, i.e., repetitive NSSI (girls and boys) and high-risk DE (girls).

Subsequently, we modelled *n* in each group *g* out of total observations at that period/wave with a stepwise binomial generalized linear model with a log link (step 1: equation 5, step 2: equation 6). We opted for the log link (rather than the logit link) so that exponentiated coefficients represented multiplicative effects on outcome probability (i.e., risk ratios) rather than effects on outcome odds.

$\log(p_{g})=b_{0}+\text{ }b_{1}(\text{background)}_{g}+b_{2}(\text{wave)}_{g}+b_{3}(\text{period)}_{g}+b_{4}(\text{behaviour)}_{g}+b_{5}(\text{MHP)}_{g}+b_{6}(\text{behaviour}\times\text{MHP})_{g}$	(5)

$\log(p_{g})=b_{0}+\text{ }b_{1}(\text{background)}_{g}+b_{2}(\text{wave)}_{g}+b_{3}(\text{period)}_{g}+b_{4}(\text{behaviour)}_{g}+b_{5}(\text{MHP)}_{g}+b_{6}(\text{behaviour}\times\text{MHP})_{g}+b_{7}(\text{period}\times\text{behaviour})_{g}+b_{8}(\text{period}\times\text{MHP})_{g}+b_{9}(\text{period}\times\text{behaviour}\times\text{MHP})_{g}$	(6)

The models included the covariates “wave” (*Baseline: 2007, 2024* vs. *Follow-up: 2008, 2025*) and “background” (*Swedish* vs. *Foreign*), enabling proportions (research question 2) and estimates (research question 3) to be adjusted accordingly. The independent variables relevant to the research aims included “period” (*2007/2008* vs. *2024/2025*), “behaviour” (*no repetitive NSSI* vs. *repetitive NSSI*, or *no high-risk DE* vs. *high-risk DE*), and “MHP” (*below clinically relevant threshold for total MHP* vs. *at/above clinically relevant threshold for total MHP*), and their two- and three-way interactions.

***Research question 2: Combined time trends in prevalence***

The R package *emmeans* (version 1.11.2-8)^10^ was used to estimate adjusted proportions of participants scoring (1) below both thresholds, (2) at/above the behavioural measure only (repetitive NSSI/high-risk DE), (3) at/above the cutoff for MHP only, and (4) at/above both thresholds for each period, using the model specified at step 2 (equation 6). Proportions for each specific combination were then compared between periods with a Bonferroni-corrected *z*-test, enabling us to test the magnitude of change within each group.

***Research question 3: Time trends in interconnectivity***

Research question 3 centred on the *behaviour* interaction at step 1 (equation 5), and the *behaviour* and *period×behavior×MHP* interactions at step 2 (equation 6). At step 1, the two-way interaction estimates the average strength of the association between the behaviour to clinically relevant MHP, regardless of period. At step 2, the two-way interaction indicates the strength of the association between the behaviour and clinically relevant MHP in the first period, while the three-way interaction estimates the magnitude of change of that association to the second period.

**References**

1.	Little RJ. A test of missing completely at random for multivariate data with missing values. *J Am Stat Assoc* 1988; **83(404)**: 1198–202. doi:10.1080/01621459.1988.10478722

2.	Ullman JB, Bentler PM. Structural equation modeling. In: Schinka JA, Velicer WF, Weiner IB, editors. Handbook of Psychology. 2nd ed. JohnWiley & Sons; 2013: 661–90. doi:10.1002/9781118133880.HOP202023

3.	Van Buuren S, Groothuis-Oudshoorn K. mice: Multivariate imputation by chained equations in R. *Journal Stat Softw* 2011; **45**: 1–67. doi:10.18637/jss.v045.i03

4.	Austin PC, van Buuren S. Logistic regression vs. predictive mean matching for imputing binary covariates. *Stat Methods Med Res* 2023; **32(11)**: 2172–83. doi:10.1177/09622802231198795

5.	Bates D, Mächler M, Bolker B, Walker S. Fitting linear mixed-effects models using lme4. *Journal Stat Softw* 2015; **67**: 1–48. doi:10.18637/jss.v067.i01

6.	Singmann H, Bolker B, Westfall J, Aust F, Ben-Shachar M. afex: Analysis of Factorial Experiments. Version number 1.5-0 [software]. 2025 [cited 2025 November 12]. Available from doi:10.32614/CRAN.package.afex

7.	Singer JD, Willnett, JB. *Applied longitudinal analysis: Modeling change and event occurrence*. Oxford University Press, Inc 2003.

8.	Liu J, Ma Y, Johnstone J. A goodness-of-fit test for zero-inflated Poisson mixed effects models in tree abundance studies. *Comput Stat Data Anal* 2020; **144**: 106887. doi:10.1016/j.csda.2019.106887

9.	Rizopoulos D. GLMMadaptive: Generalized Linear Mixed Models using Adaptive Gaussian Quadrature. Version number 0.9-7 [software]. 2025 [cited 2025 November 12]. Available from doi:10.32614/CRAN.package.GLMMadaptive

10.	Lenth R. emmeans: Estimated Marginal Means, aka Least-Squares Means. Version number 1.11.2-8 [software]. 2025 [cited 2025 November 12]. Available from doi:10.32614/CRAN.package.emmeans
